# Cognitive screening performance in patients with inflammatory bowel disease in remission: a case-control study

**DOI:** 10.1093/crocol/otag108

**Published:** 2026-10-29

**Authors:** Costantino Sgamato, Carmen Ambrosio, Stefano Minieri, Giovanni Zerella, Alba Rocco, Pietro Coccoli, Gerardo Nardone, Debora Compare

**Affiliations:** Department of Clinical Medicine and Surgery, Gastroenterology, University Federico II, Naples, Italy; Department of Clinical Medicine and Surgery, Gastroenterology, University Federico II, Naples, Italy; Department of Clinical Medicine and Surgery, Gastroenterology, University Federico II, Naples, Italy

**Keywords:** gut–brain axis, cognitive impairment, extraintestinal manifestations, inflammatory bowel disease

## Abstract

**Background:**

Emerging evidence links inflammatory bowel disease (IBD) to neurological and mood disorders, but cognitive function in this population remains understudied. By using Montreal Cognitive Assessment (MoCA) we screened for mild cognitive impairment (MCI) patients with IBD and healthy controls (HC).

**Methods:**

We enrolled a highly selected cohort of 120 patients with IBD in clinical, biochemical, and endoscopic remission (59 with ulcerative colitis [UC] and 61 with Crohn’s disease [CD]) and 115 matched HC. A MoCA score <26 was considered a below-threshold cognitive screening result, not a clinical diagnosis of mild cognitive impairment. The Hospital Anxiety and Depression Scale (HADS) was used to assess current symptoms rather than establish psychiatric diagnoses.

**Results:**

MoCA scores <26 were more frequent among patients with IBD than HC (57% vs. 42%, *P* = .016), even after excluding participants with HADS scores in the probable-symptom range (57.9% vs. 37.5%, *P* = .009). Mean total MoCA scores did not differ significantly between groups (25.0 vs. 25.3, *P* = .280). In exploratory subgroup comparisons, patients with UC had lower naming-domain scores than HC (*P* = .002). In multivariable analysis, age (odds ratio [OR] 1.03, 95% confidence interval [CI] 1.0–1.1; *P* = .002), IBD diagnosis (OR 3.07, 95% CI 1.4–6.4; *P* = .003), and years of schooling (OR 0.85, 95% CI 0.7–1.0; *P* = .030) were independently associated with a MoCA score <26. After excluding participants with HADS scores in the probable-symptom range, IBD diagnosis (OR 2.72, 95% CI 1.1–6.5; *P* = .025) and years of schooling (OR 0.71, 95% CI 0.6–0.9; *P* = .001) remained associated with a below-threshold score.

**Conclusions:**

In this highly selected sample, IBD was associated with a greater proportion of below-threshold MoCA scores. These findings reflect cognitive performance and do not establish a higher prevalence of cognitive impairment, a causal mechanism, or a rationale for universal screening. Longitudinal studies incorporating diagnostic neuropsychological assessment are needed.

## Graphical Abstract

**Graphical Abstract:**
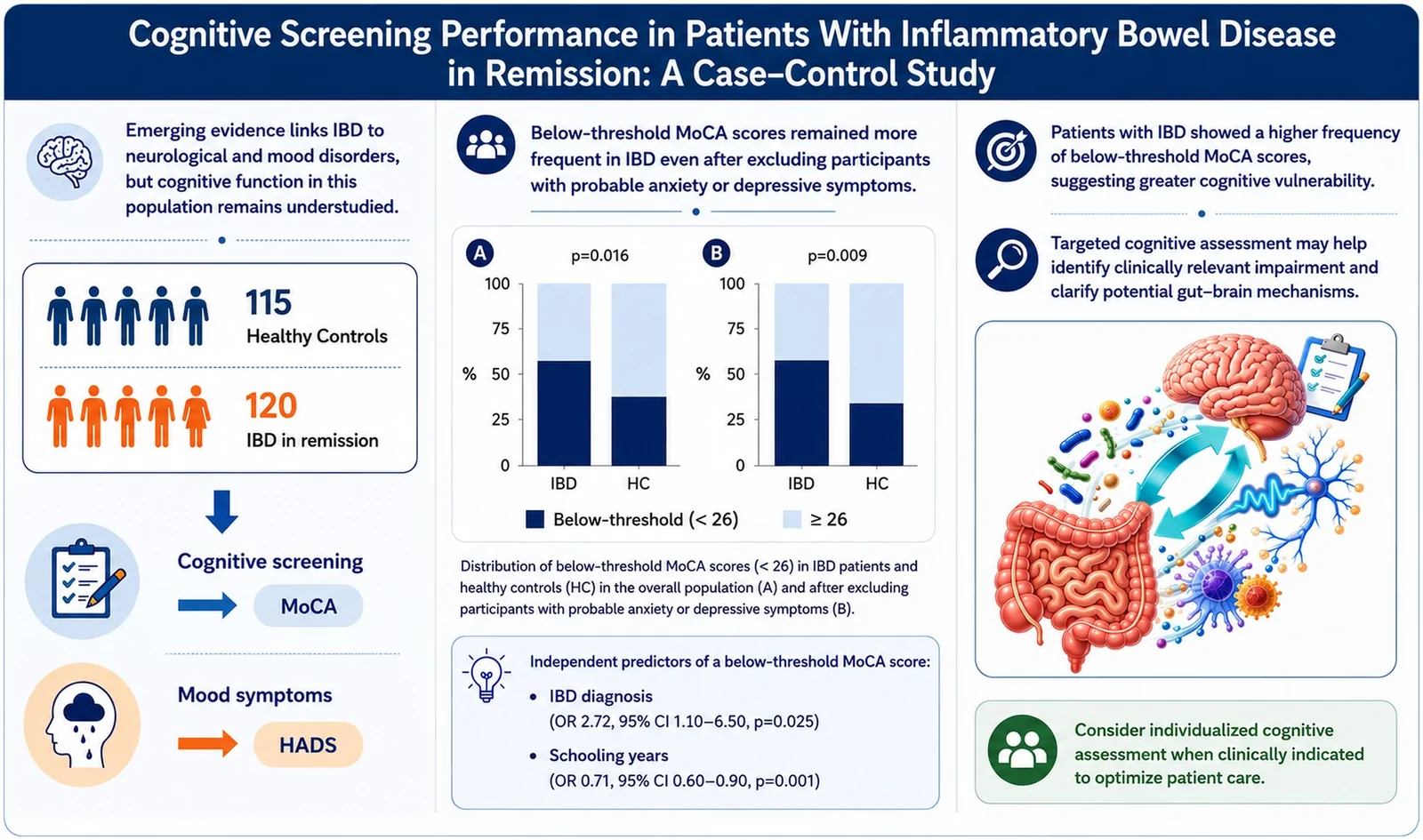

## Introduction

Inflammatory bowel diseases (IBD), consisting mainly of ulcerative colitis (UC) and Crohn’s disease (CD), are chronic inflammatory disorders, characterized by recurrent periods of disease activity and remission. The annual incidence rates are increasing worldwide, especially in countries undergoing industrialization, ranging from 10.5 to 46.14 per 100 000 in Europe, 1.37 to 1.5 per 100 000 in Asia and the Middle East, 23.67 to 39.8 per 100 000 in Oceania, 0.21 to 3.67 per 100 000 in South America, and 7.3 to 30.2 per 100 000 in North America.^1^

The pathogenesis of IBD is still largely undefined, but it is assumed to result from a dysregulated immune response to environmental factors in genetically susceptible individuals. IBDs are accompanied by multiple extraintestinal manifestations, mainly joint and eye disease.^2^ However, compelling evidence suggests that IBD can also impact brain function and structure. Being affected by IBD increases the risk of developing mood and behavioral disorders as well as neurological diseases, including dementia.^3–6^ Furthermore, a growing number of neuroimaging studies showed structural and functional abnormalities in people with IBD.^7–9^ For example, neurological imaging in IBD has revealed grey matter alterations in regions linked to information processing and memory, diffuse white matter hyperintensities, and changes in global network organization and regional connectivity.^10^

These findings support a bidirectional communication between the gut (and its microbiome) and the central nervous system (gut–brain axis).^11^

Cognitive deficits in IBD are plausible, as chronic inflammation appears to have a cumulative detrimental impact on neuroinflammation, neuronal loss, and neurotransmission.^12^ Furthermore, inflammation can modify synaptic plasticity and neuronal activity, especially in the hippocampus, a critical brain region implicated in cognitive processes and mood regulation.^13^ Disruptions of the intestinal epithelial barrier and gut microbiome dysbiosis may facilitate the passage of microbiome-derived neurotoxic metabolites into the central nervous system.^14^ Finally, deficiencies of micronutrients such as vitamin B12 and D, commonly observed in IBD patients, are associated with cognitive impairment in the general population.^15^^,^^16^

Mild cognitive impairment (MCI) is a heterogeneous clinical syndrome characterized by a decline in cognitive abilities greater than expected for age, but not severe enough to substantially interfere with independence in everyday activities. It may involve memory, language, decision-making, attention, or other cognitive domains and can be associated with subsequent cognitive and functional decline. Importantly, a clinical diagnosis of MCI requires a comprehensive assessment that considers objective testing, subjective concerns, functional status, medical and psychiatric factors, and, where appropriate, formal neuropsychological evaluation; it cannot be established by a single screening cutoff.^17^^,^^18^

A few studies have examined cognitive performance or possible MCI in IBD patients, yielding conflicting results because of small sample sizes and heterogeneity in assessment methods and enrolled populations. Moreover, whether and to what extent IBD activity is associated with cognitive performance remains insufficiently explored.^19–22^

Based on evidence linking chronic inflammation and chronic illness to cognitive dysfunction, we hypothesized that patients with IBD in clinical remission would more frequently have MoCA scores below the conventional screening threshold than demographically matched healthy controls (HC). Given prior reports of structural and functional brain alterations in regions involved in memory and information processing, we further anticipated that memory and executive functions might be affected. However, because of heterogeneity in the existing literature, analyses of individual cognitive domains and IBD subtype were considered exploratory. Accordingly, this study aimed to screen for cognitive performance patients with IBD in clinical remission and HC.

## Methods

### Patients’ characteristics

This was a prospective, cross-sectional case-control study.

We enrolled consecutive adult patients (18–65 years) with UC or CD attending a tertiary care center from February 1, 2025 to May 15, 2025. Participants were interviewed, and their medical records were reviewed to obtain data regarding disease duration, type of therapy, presence of extraintestinal manifestations, and past complications (eg, bowel obstruction or fistula formation).

We included IBD patients who had undergone an ileocolonoscopy within the 6 months before enrollment. Endoscopic remission was defined as SES-CD <3 points or absence of ulcerations for CD and Mayo endoscopic subscore = 0 points for UC, respectively. As far as laboratory data, we enrolled patients with C-reactive protein (CRP) under the upper limit of normal and fecal calprotectin (FC) level below 150 µg/g in the month preceding enrollment. Clinical remission was defined as Harvey–Bradshaw Index <5 for CD and Partial Mayo score <3 and no score >1, for UC, respectively.^23^

Healthy volunteers with at least an elementary school education were recruited as HC. For both IBD patients and HC, exclusion criteria included a previously established psychiatric diagnosis (including anxiety or depression), neurological disease with established cognitive sequelae (eg, stroke or psychotic disorder), language difficulties, sleep disorders, ileostomy or colostomy, severe comorbidities (advanced renal, hepatic, cardiac, pulmonary, and/or thyroid disorders), alcohol abuse (>40 g of alcohol per day on average), and use of corticosteroids or other medications with known effects on neuropsychological function during the 3 months before enrollment. Thus, the psychiatric exclusion criterion referred to a preexisting clinical diagnosis. Sleep disorders were screened using the Pittsburgh Sleep Quality Index (PSQI), which assesses subjective sleep quality, sleep latency, duration, efficiency, disturbances, medication use, and daytime dysfunction; a global PSQI score >5 indicated poor sleep.^24^

### Assessment of cognitive function and mood disorders

Cognitive function was assessed in all participants using the Italian version of the Montreal Cognitive Assessment (MoCA), a one-page, 30-point screening test administered in approximately 10 minutes. Visuospatial abilities were assessed with clock drawing (3 points) and 3-dimensional cube copy (1 point). Executive functions were evaluated using an alternation task adapted from Trail Making Test B (1 point), phonemic fluency (1 point), and 2-item verbal abstraction (2 points). Language was assessed using 3-item confrontation naming (lion, rhinoceros, camel; 3 points), repetition of 2 syntactically complex sentences (2 points), and fluency. Short-term memory was assessed by 2 learning trials of 5 nouns and delayed recall after approximately 5 minutes (5 points). Attention, concentration, and working memory were evaluated using sustained attention (1 point), serial subtraction (3 points), and forward and backward digit span (1 point each). Orientation to time and place was evaluated (6 points). Higher total scores indicate better screening performance. A score <26 was classified as a below-threshold screening result for the present analysis. The education correction of 1 additional point for participants with ≤12 years of education was applied. The MoCA is a screening instrument; a score <26 alone was not considered diagnostic of MCI. Anxiety and depressive symptoms were assessed with the Hospital Anxiety and Depression Scale (HADS), a 14-item self-report screening questionnaire with separate anxiety (HADS-A) and depression (HADS-D) subscales ranging from 0 to 21. Scores ≥8 and >11 were described as possible- and probable-symptom ranges, respectively. These thresholds identify symptom burden and do not establish a psychiatric diagnosis.^25^

### Statistical analysis

This study is designed as a proof-of-concept investigation; therefore, a formal sample size calculation is not required. Continuous variables were expressed as mean ± standard deviation (SD) or median and interquartile range (IQR) according to their distribution, assessed with the Kolmogorov–Smirnov test. Frequencies were calculated for categorical variables. The Mann–Whitney rank-sum test or Kruskal–Wallis test was used to compare continuous variables. The chi-square (χ²) and Fisher’s exact test were used to compare the categorical variables. The difference in the proportion of individuals with a MoCA score below 26 between IBD patients and HCs was assessed using a χ² or Fisher’s exact test. A univariate analysis was performed to establish whether baseline clinic psychometrics parameters contributed to predicting MoCa score below the lower limit of normal. Multivariate analysis was then performed using logistic regression, which calculated the odds ratio (OR) and a confidence interval (CI) of 95%. A *P*-value <0.05 was taken as the level of significance. All statistical procedures were performed using SPSS version 22.0 for Windows (SPSS Inc., Chicago, IL). The artwork was created by using GraphPad Prism 5.0.

### Ethical considerations

The present study conforms to the ethical guidelines of the Declaration of Helsinki. The study was approved by the relevant Institutional Review Board (approval number 99/2025). Written informed consent was obtained from all participants.

## Results

Among 291 consecutive IBD patients assessed for eligibility, 171 were excluded because of active disease (*n* = 52), use of non-permitted medications in the 90 days before enrollment (*n* = 26), a previous diagnosis of a mood or other psychiatric disorder (*n* = 16), major bowel surgery (*n* = 21), sleep disorders (*n* = 9), alcohol abuse (*n* = 10), severe comorbidities (*n* = 18), or refusal to participate (*n* = 19). The resulting IBD sample therefore represented 41% of the consecutive patients assessed and was restricted to individuals in remission without several common clinical factors that may affect cognitive performance.

Finally, 120 IBD patients (59 UC and 61 CD) and 115 HC were enrolled in the study and considered for statistical analysis. The mean age of the HC group was 45.9 ± 16.6 years, whereas in the IBD group, it was 42.9 ± 14.6 years. None of the demographic variables (gender, age, BMI, smoking habits, marital status, residence, and years of schooling) significantly differed between the groups (Table 1).

**Table 1:** Clinicopathological characteristics of the enrolled cohort stratified based on disease status.

	**HC *n*** **=** **115**	**IBD *n*** **=** **120**	*P*
**Males (*n*. %)**	51 (44)	63 (52)	.211
**Age (years, mean** ± **SD)**	45.9 ± 16.6	42.9 ± 14.6	.231
**BMI (kg/m^2^. mean** ± **SD)**	25.4 ± 4.1	25.3 ± 3.9	.657
**Smoking (*n*. %)**			
**- Never**	72 (62.6)	78 (65)	.088
**- Active**	34 (29.5)	20)	
**- Ex-smoker**	9 (7.8)	18 (15)	
**Married (*n*. %)**	71 (61.7)	68 (56.6)	.874
**Residence**			
**Urban**	101 (88)	93 (77)	.164
** Suburban**	14 (12)	22 (18)	
** Rural**	0	5 (5)	
**Moderate alcohol users**	3 (2.6)	2 (1.6)	.787
**Years of schooling (y. median [IQR])**	13 [11–13]	13 [10–13]	.348
**MoCA Score (mean** ± **SD)**	25.3 ± 2.6	25 ± 2.6	.280
**<26 (*n*. %)**	48 (42)	69 (57)	.016
**HADS-A score (mean** ± **SD)**	8.3 ± 4.6	8.4 ± 4.7	.780
** HADS-A** **>** **11 (*n*. %)**	35 (30.4)	31 (25.8)	.433
**HADS-D score (mean** ± **SD)**	4.9 ± 3.5	5.3 ± 3.7	.356
** HADS-D** **>** **11 (*n*. %)**	6 (5.2)	12 (10)	.168
**Sensitivity analysis^a^**			
**<26 (*n*. %)**	30(37.5)	51(57.9)	.009

Abbreviations: BMI, body mass index; HADS-A, Hospital Anxiety and Depression Scale—Anxiety subscale; HADS-D, Hospital Anxiety and Depression Scale—Depression subscale; HC, healthy controls; IBD, inflammatory bowel disease; MoCA, Montreal Cognitive Assessment.

a Sensitivity analysis excluded participants with HADS-A or HADS-D > 11 (HC *n* = 80; IBD *n* = 88); HADS is not diagnostic.

Overall, 117 participants (48 HC and 69 IBD patients) had a MoCA score <26. The proportion of individuals with a MoCA score <26 was significantly higher among IBD patients than HC (57% vs. 42%; *P* = .016). The absolute difference was 15.8% (95% CI 3.0%–28.6%), and the relative prevalence of a below-threshold score was 1.38 (95% CI 1.06–1.80). After excluding participants with HADS scores in the probable-symptom range for anxiety or depression, a below-threshold MoCA score remained more frequent in IBD patients than HC (57.9% vs. 37.5%; *P* = .009). The absolute difference was 20.5% (95% CI 5.3%–35.6%), with a relative prevalence of 1.55 (95% CI 1.11–2.16) (Figure 1). Mean total MoCA scores, however, did not differ significantly between IBD patients and HC (25.0 ± 2.6 vs. 25.3 ± 2.6, *P* = .280; Table 1).

**Figure 1:**
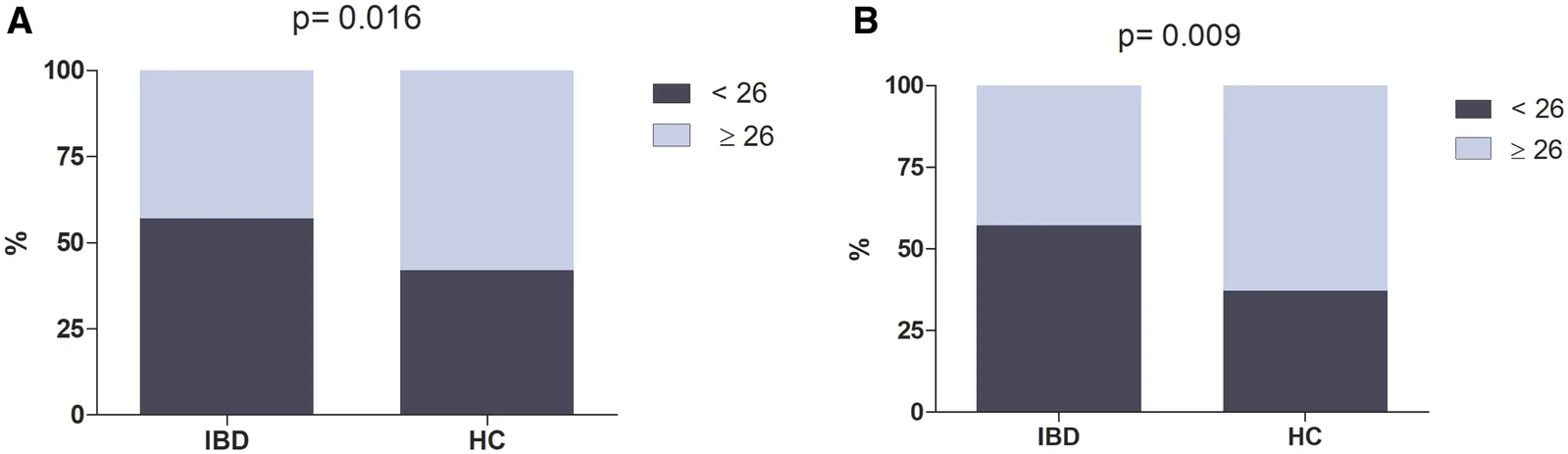
Distribution of Montreal Cognitive Assessment (MoCA) scores below the conventional screening threshold (<26) for mild cognitive impairment (MCI) among inflammatory bowel disease (IBD) patients and healthy controls (HC) in the overall population (A) and after excluding participants with Hospital Anxiety and Depression Scale (HADS) scores in the probable-symptom range for anxiety or depression (B).

The MoCA score inversely correlated with age (*r* = −0.4, *P* < 0.001), anxiety (*r* = −0.2, *P* = .011), and depression score (*r* = −0.1, *P* = .044) and directly correlated with years of schooling (*r* = 0.4, *P* < 0.001). In the subset of IBD patients, the MoCA score was inversely correlated with age (*r* = −0.39, *P* < 0.001) and depression (*r* = −0.2, *P* = .007) and positively correlated with years of schooling (*r* = 0.4, *P* < 0.001).

In pairwise exploratory comparisons of HC with CD and UC, CD patients were younger (45.9 vs. 40.1 years, *P* = .020), had lower BMI (25.4 vs. 24.1 kg/m^2^, *P* = .037), and less often resided in an urban area (88% vs. 70%, *P* = .047) than HC (Table 2). The proportion with a MoCA score <26 did not differ between CD and HC (46% vs. 42%; *P* = .633); the absolute difference was 4.2% (95% CI −11.2% to 19.5%) and the prevalence ratio was 1.10 (95% CI 0.78 − 1.56).

**Table 2:** Clinicopathological characteristics of patients with UC and CD compared to healthy HC, respectively.

	**HC *n*** **=** **115**	**UC *n*** **=** **59**	*P*	**CD *n*** **=** **61**	*P*
**Males (*n*. %)**	51(44)	27 (45.7)	.859	36 (59)	.064
**Age (years, mean** ± **SD)**	45.9 ± 16.6	45.9 ± 14.7	.982	40.1 ± 14.1	.020
**BMI (kg/m^2^. mean** ± **SD)**	25.4 ± 4.1	26.56 ± 4.6	.073	24.1 ± 3.6	.037
**BMI** **>** **30 (*n*. %)**	14 (12.1)	9 (15.2)	.570	4 (6.5)	.242
**Smoking (*n*. %)**					
** Never**	72 (62.6)	38 (64.4)	.677	40 (65.5)	.741
**Active**	34 (29.5)	9 (15.2)		15 (24.5)	
** Ex-smoker**	9 (7.8)	12 (20.3)		6 (9.8)	
**Married (*n*. %)**	71 (61.7)	37 (62.7)		31 (50.8)	.688
**Residence**					
** Urban**	101(88)	50 (84)	.845	43 (70)	.047
** Suburban**	14(12)	8 (14)		14 (23)	
** Rural**	0	1 (2)		4 (7)	
**Moderate alcohol users**	3 (2.6)	1 (1.7)		1 (1.63)	.782
**Years of schooling (y. median [IQR])**	13 [11–13]	13 [8–13]	.093	13 [7–13]	.881
**MOCA score (mean** ± **SD)**	25.3 ± 2.6	24.4 ± 2.86	.026	25.6 ± 2.3	.610
** <26 (*n*. %)**	48(42)	41 (69)	<0.001	28 (46)	.633
**HADS-A score (mean** ± **SD)**	8.3 ± 4.6	8.8 ± 4.9	.511	8.1 ± 4.5	.841
** HADS-A** **>** **11 (*n*. %)**	35 (30.4)	19 (32.2)	.811	12 (19.6)	.125
**HADS-D score (mean** ± **SD)**	4.9 ± 3.5	5.4 + 3.6	.332	5.2 + 3.8	.572
** HADS-D** **>** **11 (*n*. %)**	6 (5.2)	6 (10.1)	.222	6 (9.8)	.247
**Sensitivity analysis^a^**					
** <26 (*n*. %)**	30 (37.5)	27(67)	.003	24(50)	.197

Abbreviations: BMI, body mass index; CD, Crohn’s disease; HADS-A, Hospital Anxiety and Depression Scale—Anxiety subscale; HADS-D, Hospital Anxiety and Depression Scale—Depression subscale; HC, healthy controls; MoCA, Montreal Cognitive Assessment; UC, ulcerative colitis.

a Sensitivity analysis excluded participants with HADS-A or HADS-D > 11 (HC *n* = 80; UC *n* = 40; CD *n* = 48); HADS is not diagnostic.

In the exploratory UC-HC comparison, a MoCA score <26 was more frequent in UC than HC (69% vs. 42%; *P* = .0007). The absolute difference was 27.8% (95% CI 12.1%−43.4%), and the prevalence ratio was 1.66 (95% CI 1.27 − 2.19). This subgroup result should be interpreted cautiously because it was not the primary analysis and potential confounding was not fully controlled.

After excluding participants with HADS scores in the probable-symptom range for anxiety or depression, a MoCA score <26 remained more frequent in UC than HC (67% vs. 37.5%; *P* = .003), with an absolute difference of 30% (95% CI 11%−49%) and prevalence ratio of 1.80 (95% CI 1.26 − 2.57) (Figure 2). The corresponding CD-HC comparison was not significant (50% vs. 37.5%; *P* = .197; absolute difference 12.5%, 95% CI −5.2% to 30.2%; prevalence ratio 1.33, 95% CI 0.89 − 1.99). In direct UC-CD comparisons, UC patients were older (45.9 vs. 40.1 years, *P* = .029), had higher BMI (26.5 vs. 24.1 kg/m^2^, *P* = .001), lower mean MoCA scores (24.4 vs. 25.6, *P* = .017), and a greater proportion of MoCA scores <26 (69% vs. 46%, *P* = .035); CD patients more frequently had ongoing or former biologic exposure (85% vs. 56%, *P* < 0.001) (Table 3). These differences, together with potential unmeasured disease-related factors, limit causal or disease-specific interpretation of the subgroup findings.

**Figure 2:**
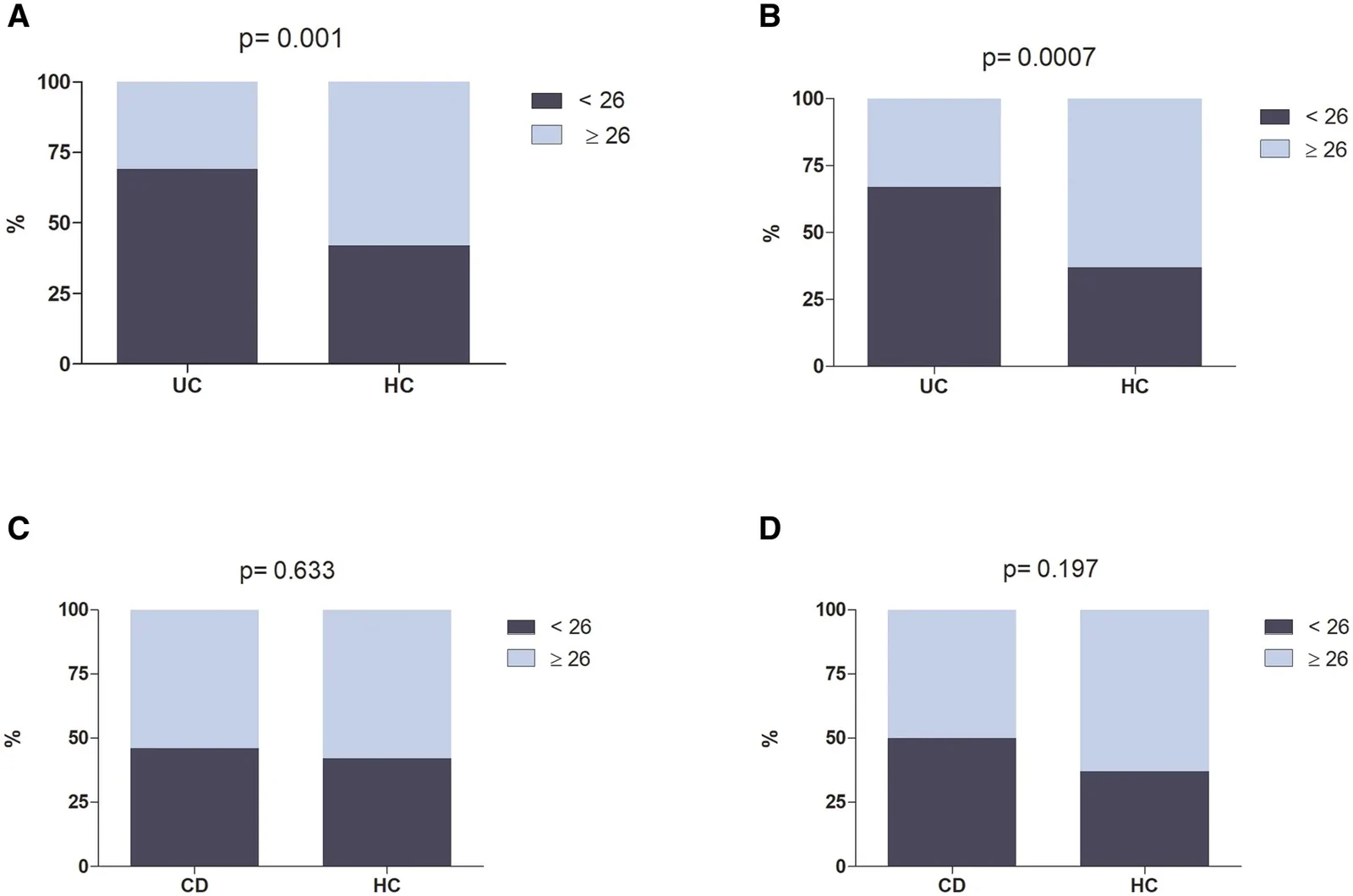
Distribution of Montreal Cognitive Assessment (MoCA) scores below the conventional screening threshold (<26) for mild cognitive impairment (MCI) in patients with ulcerative colitis (UC, panel A, B) and Crohn's disease (CD, panel C, D). Panel A and C include all patients; B and D exclude those with HADS scores in the probable-symptom range for anxiety or depression. Subgroup analyses were exploratory.

**Table 3:** Clinicopathological characteristics of the population with inflammatory bowel disease.

	**UC *n*** **=** **59**	**CD *n*** **=** **61**	*P*
**Males (*n*. %)**	27 (45.7)	36 (59)	.146
**Age (years, mean** ± **SD)**	45.9 ± 14.7	40.1 ± 14.1	.029
**BMI (kg/m^2^. mean** ± **SD)**	26.56 ± 4.6	24.1 ± 3.6	.001
**BMI** **>** **30 (*n*. %)**	9 (15.2)	4 (6.5)	.125
**Smoking (*n*. %)**			
**Never**	38 (64.4)	40 (65.5)	.172
**Active**	9 (15.2)	15 (24.5)	
**Ex-smoker**	12 (20.3)	6 (9.8)	
**Married (*n*. %)**	37 (62.7)	31 (50.8)	.246
**Residence**			
** Urban**	50 (84)	43 (70)	.277
** Suburban**	8 (14)	14 (23)	
** Rural**	1 (2)	4 (7)	
**Moderate alcohol users (*n*. %)**	1 (1.7)	1 (1.63)	
**Years of schooling (y. median [IQR])**	13 [8-13]	13 [7-13]	.104
**MoCA Score (mean** ± **SD)**	24.4 ± 2.86	25.6 ± 2.3	.017
** < 26 (*n*. %)**	41 (69.5)	28 (46)	.009
**HADS-A score (mean** ± **SD)**	8.8 ± 4.9	8.1 ± 4.5	.455
** HADS-A** **>** **11 (*n*. %)**	19 (32.2)	12 (19.6)	.177
**HADS-D score (mean** ± **SD)**	5.4 ± 3.6	5.2 ± 3.8	.741
** HADS-D** **>** **11 (*n*. %)**	6 (10.1)	6 (9.8)	.951
**Sensitivity analysis^a^**	27 (67)	24 (50)	.098
**Disease characteristics**			
**Time since diagnosis (y, mean** ± **SD)**	12.3 ± 8.8	9.8 ± 7.5	.109
**Age at diagnosis (median [IQR])**	31.1 [23-46]	26.4 [21-36]	.093
**Ongoing/Former Biological Therapy**	33 (56)	52 (85.2)	.001
**Extraintestinal manifestation**	8 (13.5)	5 (8.1)	.345

Abbreviations: BMI, body mass index; CD, Crohn’s disease; HADS-A, Hospital Anxiety and Depression Scale—Anxiety subscale; HADS-D, Hospital Anxiety and Depression Scale—Depression subscale; HC, healthy controls; MoCA, Montreal Cognitive Assessment; UC, ulcerative colitis.

a Sensitivity analysis excluded participants with HADS-A or HADS-D > 11 (UC *n* = 40; CD *n* = 48); HADS is not diagnostic.

In exploratory cognitive subdomain analyses, UC patients had lower median naming scores than HC both in the full sample and after excluding participants with HADS scores in the probable-symptom range (*P* = .002 and *P* = .008, respectively; Table 4). These analyses were not designed to establish domain-specific impairment and require replication.

**Table 4:** Montreal Cognitive Assessment (MoCA) domain scores by group in the overall cohort (A) and after excluding participants with HADS scores in the probable-symptom range for anxiety or depression (B). Continuous variables are expressed as median and interquartile range.

(A)	**HC *n*** **=** **115**	**IBD *n*** **=** **120**	*P*	**UC *n*** **=** **59**	*P*	**CD *n*** **=** **61**	*P*
**Visuospatial/executive**	4 [3–4]	4 [3–4]	.429	4 [3–4]	.974	4 [3–4]	.212
**Naming**	3 [3–3]	3 [3–3]	.037	3 [3–3]	.002	3 [3–3]	.961
**Attention**	6 [5–6]	5 [5–6]	.056	5 [5–6]	.086	5 [5–6]	.148
**Language**	3 [2–3]	3 [2–3]	.155	3 [2–3]	.108	3 [2–3]	.452
**Abstraction**	2 [1–2]	2 [2–2]	.209	2 [1–2]	.911	2 [2–2]	.052
**Memory**	3 [2–4]	3 [2–4]	.212	3 [1–4]	.052	3 [2–4]	.904
**Orientation**	6 [5–6]	6 [4–6]	.552	6 [4–6]	.676	6 [4–6]	.596

(B)	**HC *n*** **=** **80**	**IBD *n*** **=** **88**	*P*	**UC *n*** **=** **40**	*P*	**CD *n*** **=** **48**	*P*
**Visuospatial/executive**	4 [3–4]	4 [3–4]	.648	3 [3–4]	.141	4 [3–5]	.551
**Naming**	3 [3–3]	3 [3–3]	.072	3 [3–3]	.008	3 [3–3]	.714
**Attention**	6 [5–6]	5 [5–6]	.029	5 [5–6]	.059	5 [5–6]	.079
**Language**	3 [2–3]	3 [2–3]	.948	3 [2–3]	.778	3 [2–3]	.884
**Abstraction**	2 [1–2]	2 [1–2]	.564	2 [1–2]	.972	2 [2–2]	.352
**Memory**	3 [2–4]	3 [2–4]	.415	3 [2–4]	.305	3 [2–4]	.723
**Orientation**	6 [4–6]	6 [4–6]	.607	6 [4–6]	.520	6 [4–6]	.830

Abbreviations: CD: Crohn’s disease; HC, healthy controls; IBD, inflammatory bowel disease; UC, ulcerative colitis.

In univariate analysis, age (*P* = .001), BMI (*P* = .006), IBD diagnosis (*P* = .001), years of schooling (*P* = .001), and marital status (*P* = .001) were associated with a MoCA score <26. In multivariate analysis, age (OR 1.03, 95% CI 1.0 − 1.1, *P* = .002), IBD diagnosis (OR 3.07, 95% CI 1.06 − 6.4, *P* = .003), and years of schooling (OR 0.85, 95% CI 0.7 − 1.0, *P* = .030) independently predicted a below-threshold score (Table 5). After excluding participants with HADS scores in the probable-symptom range, IBD diagnosis (OR 2.72, 95% CI 1.10 − 6.50, *P* = .025) and years of schooling (OR 0.70, 95% CI 0.60 − 0.90, *P* = .001) remained independent predictors.

**Table 5:** Univariate and multivariate analyses of factors associated with a MoCA score <26 in the overall population (A) and after excluding participants with HADS scores in the probable-symptom range for anxiety or depression (B).

(A) Variable	Univariate			Multivariate		
	*P*-value	OR	95% CI	*P*-value	OR	95% CI
**Male sex**	.646	1.13	0.7–1.9			
**Age**	.001	1.04	1–1.1	.002	1.03	1–1.1
**BMI**	.006	1.10	1–1.2			
**IBD diagnosis**	.001	3.12	1.63–6.2	.003	3.07	1.4–6.4
**Years of schooling**	.001	0.78	0.7–0.9	.030	0.85	0.7–1
**Time since diagnosis**	.201	1.03	0.9–1			
**HADS-A** **>** **11**	.535	0.83	0.5–1.5			
**HADS-D** **>** **11**	.638	1.26	0.5–3.3			
**Marital status**	.001	0.45	0.2–0.9			

(B) Variable	Univariate			Multivariate		
	*P*-value	OR	95%CI	*P*-value	OR	95% CI
**Male sex**	.826	1.07	0.6–2			
**Age**	.001	1.04	1–1.06			
**BMI**	.103	1.07	1–1.2			
**IBD diagnosis**	.002	3.46	1.5–7.7	.025	2.72	1.1–6.5
**Years of schooling**	.001	0.71	0.6–0.9	.001	0.71	0.6–0.9
**Time since diagnosis**	.316	1.03	0.9–1			
**Marital status**	.003	0.36	0.2–0.7			

Abbreviations: BMI, body mass index; HADS-A, Hospital Anxiety and Depression Scale—Anxiety subscale; HADS-D, Hospital Anxiety and Depression Scale—Depression subscale; IBD, inflammatory bowel disease.

## Discussion

In this cross-sectional case-control study, patients with IBD in clinical, biochemical, and endoscopic remission were more likely to have a MoCA score below the conventional threshold of 26 than matched HC. This association persisted after adjustment for age and years of schooling and after excluding participants with HADS scores in the probable-symptom range. However, mean total MoCA scores were similar between the 2 groups (25.0 vs. 25.3, *P* = .280). Taken together, these findings suggest a difference in the distribution of cognitive performance rather than a generalized reduction in mean cognitive scores. Because the MoCA is a screening instrument, a score below 26 should not be considered equivalent to a clinical diagnosis of MCI.

Previous studies have also suggested an association between IBD and cognitive performance. Attree et al. first reported poorer verbal functioning among adults with IBD compared with HC.^26^ Similarly, Dancey et al. identified lower verbal intelligence quotient scores in patients with IBD than in individuals with irritable bowel syndrome or HC.^27^ A large population-based study subsequently found that IBD was associated with earlier onset and an approximately 2.5-fold higher risk of incident dementia, including Alzheimer’s disease.^4^ Although differences in study populations, disease activity, sample size, and cognitive measures limit direct comparisons, these observations support further investigation of cognitive health across the course of IBD.

Anxiety and depression are particularly relevant when cognitive performance is assessed in IBD. Both conditions may influence attention, memory, processing speed, and subjective cognitive complaints,^28–30^ and are more common in patients with IBD than in the general population.^31–33^ They also affect disease perception, daily functioning, quality of life, and patient-reported outcomes, even when intestinal disease is clinically inactive.^34^ In our study, participants with an established psychiatric diagnosis were excluded, whereas HADS was administered at enrollment to identify current anxiety and depressive symptoms. Thus, HADS scores reflected symptom burden rather than formal psychiatric diagnoses. The persistence of the association after excluding participants with scores in the probable-symptom range suggests that current anxiety or depressive symptoms did not fully explain the difference in MoCA screening results.

Disease activity may affect cognition through several interrelated pathways, including systemic inflammation, neuroendocrine responses, psychological distress, fatigue, and bidirectional gut–brain signaling.^3^ In a cross-sectional study of 105 patients with CD, Golan et al. reported subjective cognitive complaints in almost half of the participants and objective impairment in at least 1 cognitive domain in 37%, particularly affecting attention, executive function, and memory. Cognitive performance was also associated with disease activity.^20^ Similarly, Castaneda et al. found mild verbal-memory deficits in adolescents with active IBD, particularly among those receiving corticosteroids.^35^ Longitudinal evidence also indicates that active disease is associated with the subsequent development of anxiety and depressive symptoms.^3^^,^^36^

By including only patients in clinical, biochemical, and endoscopic remission, our study reduced the potential influence of concurrent intestinal activity. The observed association may therefore indicate that cognitive screening differences are not confined to periods of active disease. Nevertheless, enrollment does not exclude the possible effects of previous inflammatory activity, cumulative disease burden, prior corticosteroid or other treatment exposure, fatigue, anemia, nutritional deficiencies, or other consequences of chronic illness. These factors deserve direct evaluation in longitudinal studies.

The gut–brain axis offers a plausible conceptual framework for interpreting the relationship between IBD and cognitive performance. Intestinal inflammation and alterations in immune, neural, endocrine, and microbial pathways may influence brain function, while psychological and neurological processes may in turn modulate gastrointestinal symptoms and inflammation.^3^^,^^37^ Previous studies have reported associations between microbiota profiles and psychological symptoms in UC,^38^ as well as persistent microbiota alterations during clinical remission.^39^ These observations provide biologically plausible hypotheses but cannot establish the pathway underlying our results, as microbiome composition, inflammatory mediators, neuroimaging findings, and other mechanistic markers were not assessed in the present study.

In exploratory subgroup analyses, UC patients were more likely than HC or CD patients to have a MoCA score below 26. A previous small cross-sectional study similarly reported lower MoCA performance in patients with UC in remission than in HC and patients with irritable bowel syndrome.^40^ However, the UC and CD groups in our study differed in age, body mass index, and biologic exposure, and may also have differed in other disease- and treatment-related characteristics. The subgroup findings should therefore be considered hypothesis-generating and require confirmation in larger cohorts specifically designed to compare IBD phenotypes.

Previous studies have most consistently identified attention, executive function, and memory as cognitive domains potentially affected in IBD. Resting-state functional magnetic resonance imaging has shown associations between altered hippocampal or parahippocampal activity and memory performance in patients with active UC.^41^ Electrophysiological studies have also reported prolonged P300 latency, suggesting altered short-term memory processing,^42^ while a meta-analysis of 12 studies identified attention, particularly alertness, and executive function, particularly working memory, as the domains most consistently affected.^43^

In contrast, our exploratory domain analysis identified lower naming scores in patients with UC than in HC, after excluding participants with HADS scores in the probable-symptom range.

Naming tasks assess lexical retrieval and aspects of semantic memory.^44^ However, the MoCA naming subtest has a narrow score range and may be affected by ceiling effects. Moreover, the finding arose from multiple domain and subgroup comparisons. It should therefore be interpreted as a preliminary signal rather than evidence of a selective language deficit and should be examined using comprehensive neuropsychological instruments.

From a clinical perspective, our findings support greater awareness of cognitive concerns in patients with IBD but do not provide sufficient evidence for universal routine MoCA screening. Cognitive assessment may be considered when prompted by subjective complaints, observed functional changes, older age, substantial cumulative disease or treatment burden, or clinician concern. Potentially reversible contributors, including mood symptoms, fatigue, sleep disturbance, medication effects, anemia, and nutritional deficiencies, should also be assessed. When the MoCA is used, a below-threshold score should be communicated as a screening result rather than a diagnosis and, when clinically appropriate, followed by repeat assessment or formal neuropsychological and medical evaluation. This approach may help integrate cognitive health into multidisciplinary IBD care while avoiding premature labeling or unnecessary anxiety.

Several limitations should be considered. First, the cross-sectional design precludes conclusions about causality or cognitive changes over time. Second, the study population was highly selected: only 120 of 291 consecutive patients assessed were included, all were aged 18–65 years, attended a tertiary center, and were in clinical, biochemical, and endoscopic remission. The exclusion of active disease, major surgery, sleep disorders, established psychiatric diagnoses, severe comorbidities, recent corticosteroid exposure, and other potentially relevant conditions also resulted in an unusually IBD group. The findings may therefore not be directly generalizable to the broader IBD population or the general population, and the observed proportions should not be interpreted as prevalence estimates. Third, the MoCA does not establish a diagnosis of MCI, and comprehensive neuropsychological assessment and functional evaluation were not performed. Finally, cumulative corticosteroid exposure, historical inflammatory burden, fatigue, anemia, micronutrient deficiencies, and other potentially relevant biological or treatment-related factors were not fully characterized. The UC–CD comparisons were exploratory and potentially affected by residual confounding.

In conclusion, IBD was associated with a greater proportion of MoCA scores below the conventional screening threshold in this selected cohort, although mean total scores were similar between patients and controls. These findings suggest that cognitive health warrants further investigation in IBD and may be consistent with the broader concept of gut–brain axis involvement. Longitudinal studies in representative clinical populations, incorporating diagnostic neuropsychological assessment and direct evaluation of inflammatory, nutritional, treatment-related, and microbiome-associated pathways, are needed.

## Data Availability

The data underlying this article will be shared on reasonable request to the corresponding author.
